# Targeting mTOR in myeloid cells prevents infection-associated inflammation

**DOI:** 10.1016/j.isci.2025.112163

**Published:** 2025-03-04

**Authors:** Yohana C. Toner, Jazz Munitz, Geoffrey Prevot, Judit Morla-Folch, William Wang, Yuri van Elsas, Bram Priem, Jeroen Deckers, Tom Anbergen, Thijs J. Beldman, Eliane E.S. Brechbühl, Muhammed D. Aksu, Athanasios Ziogas, Sebastian A. Sarlea, Mumin Ozturk, Zhenhua Zhang, Wenchao Li, Yang Li, Alexander Maier, Jessica C. Fernandes, Glenn A.O. Cremers, Bas van Genabeek, Joost H.C.M. Kreijtz, Esther Lutgens, Niels P. Riksen, Henk M. Janssen, Serge H.M. Söntjens, Freek J.M. Hoeben, Ewelina Kluza, Gagandeep Singh, Evangelos J. Giamarellos-Bourboulis, Michael Schotsaert, Raphaël Duivenvoorden, Roy van der Meel, Leo A.B. Joosten, Lei Cai, Ryan E. Temel, Zahi A. Fayad, Musa M. Mhlanga, Mandy M.T. van Leent, Abraham J.P. Teunissen, Mihai G. Netea, Willem J.M. Mulder

**Affiliations:** 1 BioMedical Engineering and Imaging Institute, Icahn School of Medicine at Mount Sinai, New York, NY 10029, USA; 2 Diagnostic, Molecular and Interventional Radiology, Icahn School of Medicine at Mount Sinai, New York, NY 10029, USA; 3 Department of Internal Medicine and Radboud Center for Infectious Diseases, Radboud University Medical Center, 6525 GA Nijmegen, the Netherlands; 4 Cardiovascular Research Institute, Icahn School of Medicine at Mount Sinai, New York, NY 10029, USA; 5 Cancer Research UK Cambridge Institute, University of Cambridge, Cambridge CB2 0RE, UK; 6 Epigenomics & Single Cell Biophysics Group, Department of Cell Biology, FNWI, Radboud Institute for Molecular Life Sciences (RIMLS), Radboud University, 6525 GA Nijmegen, the Netherlands; 7 Department of Computational Biology of Individualised Medicine, Centre for Individualised Infection Medicine (CiiM), a joint venture between the Hannover Medical School and the Helmholtz Centre for Infection Research, 30625 Hannover, Germany; 8 TWINCORE, Centre for Experimental and Clinical Infection Research, a joint venture between the Hannover Medical School and the Helmholtz Centre for Infection Research, 30625 Hannover, Germany; 9 Department of Cardiology and Angiology, Heart Center Freiburg University, Faculty of Medicine, University of Freiburg, 79106 Freiburg, Germany; 10 Trained Therapeutix Discovery, 5349 AB Oss, the Netherlands; 11 SyMO-Chem B.V., 5612 AZ Eindhoven, the Netherlands; 12 Department of Cardiovascular Medicine, Experimental Cardiovascular Immunology Laboratory, Mayo Clinic, Rochester, MN 55905, USA; 13 Laboratory of Chemical Biology, Department of Biomedical Engineering, Eindhoven University of Technology, 5612 AZ Eindhoven, the Netherlands; 14 Department of Microbiology, Icahn School of Medicine at Mount Sinai, New York, NY 10029, USA; 15 Global Health and Emerging Pathogens Institute, Icahn School of Medicine at Mount Sinai, New York, NY 10029, USA; 16 4^th^ Department of Internal Medicine, National and Kapodistrian University of Athens, 124 62 Athens, Greece; 17 Marc and Jennifer Lipschultz Precision Immunology Institute, Icahn School of Medicine at Mount Sinai, New York, NY 10029, USA; 18 Icahn Genomics Institute, Icahn School of Medicine at Mount Sinai, New York, NY 10029, USA; 19 Department of Nephrology, Radboud University Medical Center, 6525 GA Nijmegen, the Netherlands; 20 Department of Medical Genetics, Iuliu Hatieganu University of Medicine and Pharmacy, 400 349 Cluj-Napoca, Romania; 21 Department of Physiology, Saha Cardiovascular Research Center, University of Kentucky, Lexington, KY 40536, USA; 22 Department of Human Genetics, Radboud University Medical Center, 6525 GA Nijmegen, the Netherlands; 23 Department of Immunology and Metabolism, Life and Medical Sciences Institute, University of Bonn, 53115 Bonn, Germany

**Keywords:** Natural sciences, Biological sciences, Biochemistry, Immunology

## Abstract

Infections, cancer, and trauma can cause life-threatening hyperinflammation. In the present study, using single-cell RNA sequencing of circulating immune cells, we found that the mammalian target of rapamycin (mTOR) pathway plays a critical role in myeloid cell regulation in COVID-19 patients. Previously, we developed an mTOR-inhibiting nanobiologic (mTORi-nanobiologic) that efficiently targets myeloid cells and their progenitors in the bone marrow. *In vitro*, we demonstrated that mTORi-nanobiologics potently inhibit infection-associated inflammation in human primary immune cells. Next, we investigated the *in vivo* effect of mTORi-nanobiologics in mouse models of hyperinflammation and acute respiratory distress syndrome. Using ^18^F-FDG uptake and flow cytometry readouts, we found mTORi-nanobiologic therapy to efficiently reduce hematopoietic organ metabolic activity and inflammation to levels comparable to those of healthy control animals. Together, we show that regulating myelopoiesis with mTORi-nanobiologics is a compelling therapeutic strategy to prevent deleterious organ inflammation in infection-related complications.

## Highlights

• mTOR pathway is intrinsically involved in COVID-19-induced myeloid cell inflammation
• mTORi-nanobiologics reduced inflammation on *in vitro* infection/trained immunity assay
• mTORi-nanobiologics reduced systemic hyperinflammation in mouse model
• mTORi-nanobiologics reduced organ inflammation in mouse model of ARDS

## Graphical abstract

**Figure:**
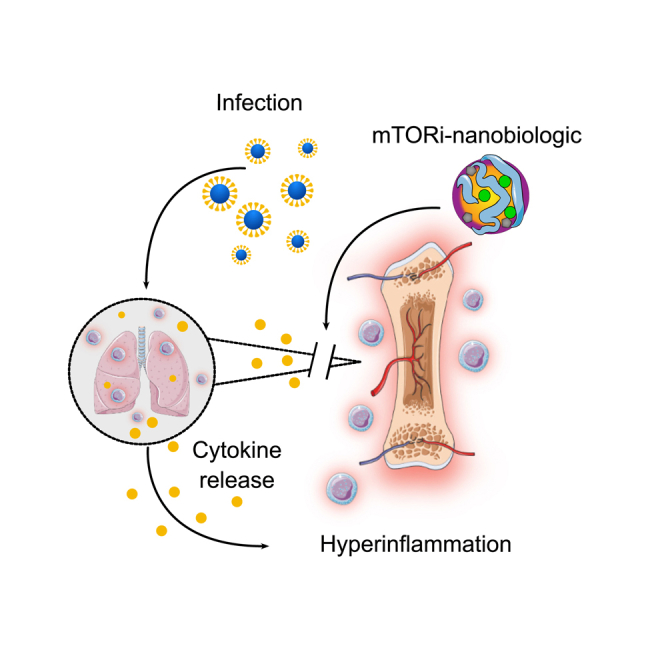

## Introduction

Exacerbated host immune response, also known as hyperinflammation, is a life-threatening condition for which limited treatment options exist.^1^ It can be triggered by diverse circumstances, including autoimmune and autoinflammatory diseases, trauma, or severe infections such as sepsis but is also observed as an adverse effect of cancer immunotherapy. It is estimated that 13.7 million patients die annually as a result of infection-related complications among which many are linked to dysregulated inflammatory responses.^2^ In these patients, immune activation becomes dysregulated resulting in exaggerated release of proinflammatory mediators such as cytokines, which subsequently spearheads endothelial cell activation, disseminated intravascular coagulation, hypotension, organ failure, and sometimes death.^1^^,^^3^ Such a hyperinflammatory response can manifest either systemically, culminating in septic shock, or locally in certain organs, such as the lungs leading to the development of acute respiratory distress syndrome (ARDS). ARDS is a prototype clinical syndrome characterized by tissue disruption, compromised gas exchange in the lungs^4^ and excessive cytokine release. It contributes to a systemic process referred to as cytokine storm, which conversely exacerbates ARDS, highlighting their interconnected nature.^5^^,^^6^^,^^7^ Currently, there are few therapeutic options available for the treatment of ARDS. In fact, poor management of ARDS is linked to mortality rates of 40% or higher in moderate-to-severe cases of hospitalized patients.^8^^,^^9^^,^^10^

Although sepsis and pneumonia drive the majority of all ARDS cases,^8^ the COVID-19 pandemic intensified research on the subject. Studies consistently report that COVID-19-triggered hyperinflammation and ARDS increase mortality.^11^^,^^12^^,^^13^^,^^14^ Current disease management strategies include alleviating symptoms and some pathophysiological approaches, such as epithelial/endothelial repair, anticoagulants, and anti-inflammatory medication.^15^ Besides the direct effects, individuals surviving ARDS often experience long-term physical and cognitive decline,^16^^,^^17^^,^^18^ further underscoring the need for innovative immunotherapeutic strategies.

While most immunotherapies for sepsis focus on the inhibition of proinflammatory cytokines,^19^ emerging research highlights the role of metabolic dysregulation in hyperinflammation. In particular, the mammalian target of rapamycin (mTOR) signaling is crucial for the activation of immune cells and is central to systemic inflammation.^20^^,^^21^^,^^22^ Sensitive to stress and inflammatory signals, mTOR controls cellular metabolism and can increase glycolytic activity by stimulating the production and activation of hexokinase, phosphofructokinase, and pyruvate kinase.^23^ Moreover, mTOR prompts hypoxia-inducible factor 1-alpha (HIF-1α) expression, which in turn promotes the release of IL-1β^24^^,^^25^ and upregulates the expression of glycolytic enzymes and glucose transporters, increasing glucose influx and usage by immune cells.^26^ This switch to glycolysis provides energy for cellular proliferation, migration, phagocytosis and production of inflammatory cytokines and mediators.^20^^,^^27^^,^^28^ Targeting mTOR in innate immune cells therefore represents an attractive approach to manage infection-associated hyperinflammation.

In this study, we used single-cell RNA sequencing (scRNA-seq) to profile peripheral blood mononuclear cells (PBMCs) from SARS-CoV-2-infected patients. In line with non-COVID-19 sepsis, we found differential expression of mTOR-related genes, especially in innate immune cells. Inspired by these findings, we evaluated our mTORi-nanobiologic^29^ in inflammation and trained immunity^30^ assays using primary human monocytes. Using the quantification of [^18^F]fluorodeoxyglucose (^18^F-FDG) uptake and flow cytometric analyses as readouts for metabolic activity and inflammation, respectively, we evaluated mTORi-nanobiologics’ *in vivo* therapeutic potential in murine models of hyperinflammation and ARDS.

## Results

### SARS-CoV-2 infection leads to differential expression of mTOR pathway genes in circulating immune cells

**Figure 1:**
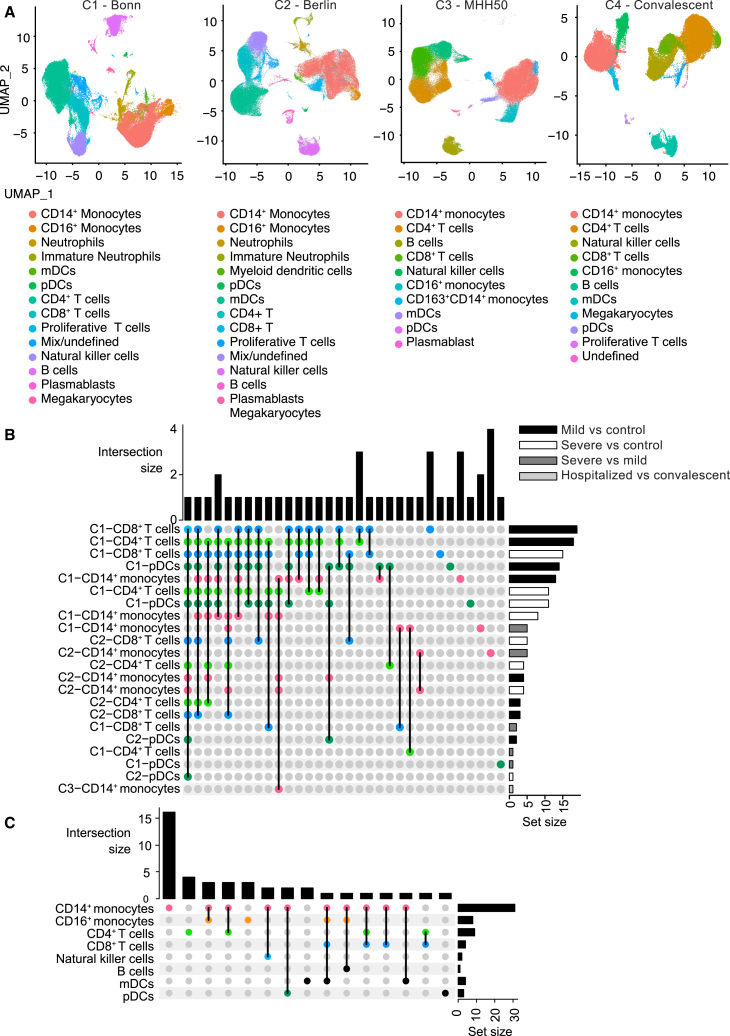
mTOR signaling pathway gene expression profiles support the use of mTORi-nanobiologics for the treatment of inflammation in COVID-19 patients (A) UMAP of scRNA-seq from the Berlin (C1), Bonn^31^ (C2), and MHH50^32^ (C3) cohorts datasets, respectively, and UMAP of scRNA-seq from convalescent COVID-19 patients^33^ (C4). (B) Upregulated differentially expressed genes across the Berlin (C1), Bonn^31^ (C2), and MHH50^32^ (C3) datasets. Intersection size represents the number of genes that were differentially expressed across the different comparisons, connected by dots, in the y axis. Set size represents the number of genes that were differentially expressed within each comparison on the y axis. (C) The number of upregulated mTOR genes in specific cell types in convalescent COVID-19 patients (C4 cohort).^33^ Intersection size represents the number of genes that were differentially expressed across different cell types, connected by dots, in the y axis. Set size represents the number of genes that were differentially expressed within each cell type on the y axis. Differentially expressed genes between every two disease conditions were identified using FindMarkers() function by Wilcoxon rank-sum test. Control = healthy volunteers, pDCs = plasmacytoid dendritic cells, mDCs = myeloid dendritic cells. See also Figures S1 and S2 and Tables S1 and S2.

We first studied mTOR signaling in scRNA-seq datasets of whole blood and PBMC samples of COVID-19 cohorts (Table S1). More precisely, we evaluated the expression profiles of 156 genes from the mTOR pathway in the circulating immune cell populations from patients with mild, severe, and convalescent COVID-19^31^^,^^32^^,^^33^ (Figure 1A, Table S2). Toward this aim, we identified cell type-specific differentially expressed genes (DEGs) from the mTOR pathway between these four independent COVID-19 patient cohorts, for which scRNA-seq data of immune cells were available. From the cohorts reported by Schulte-Schrepping et al.^31^ (Bonn and Berlin cohorts, C1 and C2, respectively), we compared healthy volunteers (control), mild, and severe COVID-19 patients for each immune cell cluster. Additionally, we compared hospitalized and convalescent participants in a different cohort (MHH50 cohort, C3) from Zhang et al.*,*^32^ as well as convalescent COVID-19 patients and healthy volunteers (control) in the Convalescent COVID-19 (C4) cohort.^33^

We identified 79 mTOR pathway-related DEGs in at least one of the analyses (Figures S1A–S1D). Compared to healthy volunteers, we found that mainly in classical monocytes from the COVID-19 patients, the mTOR pathway-related genes were differentially expressed (Figures 1B and S2A). We observed growth factor receptor bound protein 2 (GRB2), serum/glucocorticoid regulated kinase 1 (SGK1), ras homolog family member A (RHOA), and CAP-Gly domain containing linker protein 1 (CLIP1), which are associated with PI-3 kinase-mediated signal transduction, cytoskeletal modulation, and cell migration,^34^^,^^35^^,^^36^ to be upregulated in classical monocytes of different cohorts. Of note, the levels of an essential downstream effector of mTOR signaling, ribosomal subunit protein S6 (Rps6), were downregulated in most of the clusters in hospitalized or severe COVID-19 samples pointing toward decreased ribosomal biogenesis (Figures S1A–S1C). Interestingly, cells from mild and control patients differed most, while cells from mild versus severe patients showed the fewest dissimilarities. We observed the same trend in the convalescent COVID-19 cohort when focusing on upregulated DEGs, while for downregulated genes, in addition to monocytes, differences were also pronounced in natural killer cells (Figures 1C and S2B). Out of all cell types, classical and non-classical monocytes displayed the most DEGs occurring exclusively in one cell population. Our analysis of the previously published COVID-19 dataset showed that mTOR pathway-associated genes are dysregulated across various immune cell populations, albeit to a greater extent on monocyte subsets in hyperinflammatory conditions.

### Nanobiologics targeting mTOR in myeloid cells

**Figure 2:**
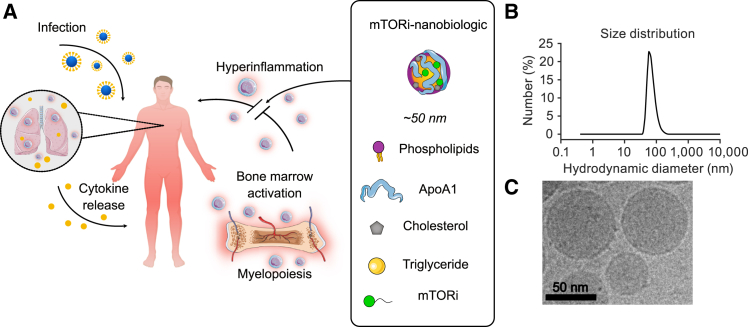
Bone marrow-avid mTORi-nanobiologics characterization (A) Study concept overview. Nanobiologics are lipoprotein-based nanoparticles with high avidity for myeloid cells and their progenitors in the bone marrow. We have loaded nanobiologics with a lipophilic prodrug of rapamycin, which is a potent mTOR inhibitor. These mTOR-inhibiting nanobiologics (mTORi-nanobiologics) dampen bone marrow activation and reduce systemic hyperinflammation upon infection. (B) mTORi-nanobiologic’s characterization by dynamic light scattering and (C) cryo-EM. Scale bar, 50 nm.

The nanobiologics platform is composed of phospholipids, cholesterol, propane-1,2,3-triyl trioctanoate, and apolipoprotein A1 (apo-A1), as we previously described.^29^ Owing to their size (of approximately 50 nm in diameter) and the apo-A1 coating, nanobiologics efficiently target myeloid cells and their hematopoietic progenitors (Figure 2A). By loading nanobiologics with an mTOR-inhibitory prodrug, the mTOR pathways can be effectively inhibited *in vivo*. We characterized the mTORi-nanobiologics’ size by dynamic light scattering (DLS, Figure 2B), while cryogenic electron microscopy reviewed spherical morphology (cryo-EM, Figure 2C).

### mTOR-inhibiting nanobiologics reduce inflammation in primary human cells *in vitro*

**Figure 3:**
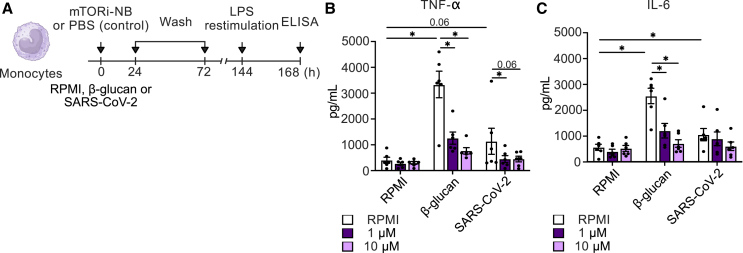
mTORi-nanobiologics reduces inflammatory response in human monocytes *in vitro* (A) Schematic representation of trained immunity assays, employed on primary monocytes derived from healthy donors. (B) *In vitro* trained immunity assay showing TNF-α production upon LPS restimulation. (C) *In vitro* trained immunity assay showing IL-6 production upon LPS restimulation. Data in (B) and (C) are presented as mean ± SEM. Statistical analysis was performed using the Wilcoxon signed-rank test. ∗*p* < 0.05. See also Figure S3 and Table S3.

We investigated the potential of mTORi-nanobiologics for the modulation of infection-associated inflammation by conducting *in vitro* experiments profiling the function of human monocytes after stimulation, in the presence or absence of nanobiologics. We previously developed a trained immunity assay^37^ that allows for the study and treatment of monocytes’ hyperresponsiveness *in vitro* (Figure 3A). Monocytes were stimulated for 24 h with β-1,3-(D)-glucan (β-glucan) or heat-inactivated SARS-CoV-2 Wuhan-Hu-1 variant^38^ or remained unstimulated in the presence or absence of 1 or 10 μM mTORi-nanobiologics. After washing the cells with warm PBS at 24 h, we washed them again at 72 h. On day 6, the cells were restimulated with 10 ng/mL *E. coli* lipopolysaccharide (LPS), and supernatant was collected 24 h later to determine tumor necrosis factor alpha (TNF-α) (Figure 3B) and interleukin-6 (IL-6) production (Figure 3C) by ELISA. TNF-α and IL-6 are gold standard markers of trained immunity, as they reflect the functional state of innate immune cells.^39^^,^^40^ We found that cells incubated with β-glucan or SARS-CoV-2 enhanced cytokine secretion upon LPS restimulation, a hyperresponsiveness indicative of innate immune memory.^30^ In contrast, cells that were treated with mTORi-nanobiologics during the initial 24-h training phase did not become hyperresponsive. A trend toward a dose-dependent effect was observed in the β-glucan group for cells treated with mTORi-nanobiologics. Similar results were also attained for trained immunity-associated pro-inflammatory chemokines (Figures S3A–S3C), where mTOR-nanobiologics treated cells showed lower CXCL-9, CXCL-10, and IL-8 production upon LPS restimulation.

### mTORi-nanobiologic therapy prevents systemic inflammation in mice

**Figure 4:**
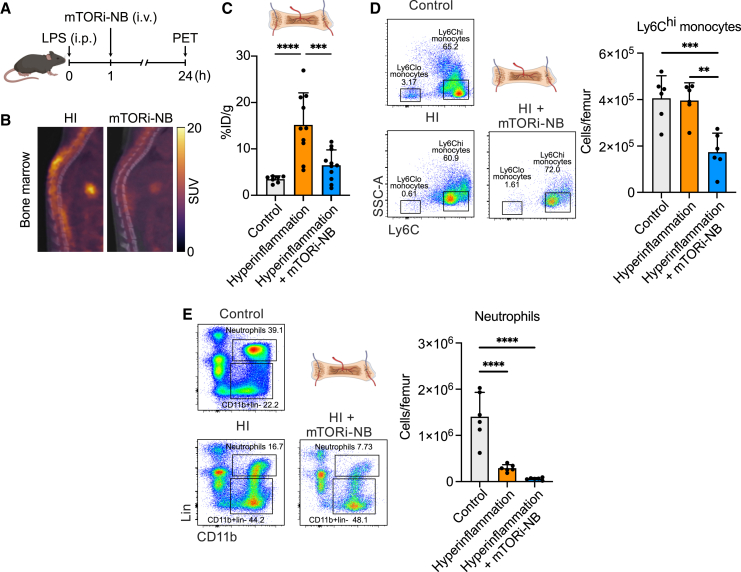
mTORi-nanobiologics reduce systemic inflammation (A) Schematic overview of the LPS-induced hyperinflammation mouse model. (B) Representative fused ^18^F-FDG PET/CT images of the spine of placebo (left panel) and mTORi-nanobiologics-treated (right panel) animals. (C) *Ex vivo* quantification of bone marrow ^18^F-FDG uptake (*n* = 7–11). (D) Representative flow cytometry plots showing Ly6C^hi^ monocytes in the bone marrow of control naive mice (negative control), mice with hyperinflammation (placebo control), and hyperinflammation mice treated with mTORi-nanobiologics, gated on live CD45^+^CD11b^+^lin^−^CD11c^−^ cells (left panel) and the associated quantification of bone marrow Ly-6C^hi^ monocytes (right panel, *n* = 6). (E) Representative flow cytometry plots showing neutrophils in the bone marrow of control naive mice (negative control), untreated hyperinflammation mice (placebo control), and hyperinflammation mice treated with mTORi-nanobiologics, gated on live CD45^+^ cells (left panel). The associated quantification of bone marrow neutrophils is also shown (right panel, *n* = 6). HI = hyperinflammation, hyperinflammation = placebo control, SUV = standardized uptake value. Data in (C)–(E) are plotted as mean ± SD. Statistical analyses were performed using the Shapiro-Wilk test, followed by one-way ANOVA (with Tukey’s multiple comparisons test) or Kruskal-Wallis (followed by Dunn’s multiple comparison test) according to Gaussian distribution. ∗∗*p* < 0.01, ∗∗∗*p* < 0.001, ∗∗∗∗*p* < 0.0001. See also Figure S4.

Critical aspects of the exaggerated systemic inflammation caused by bacterial sepsis can be mimicked in mice by administering the gram-negative bacterial component LPS.^41^^,^^42^ To investigate the effects of our mTORi-nanobiologics in regulating myelopoiesis during hyperinflammation processes, we intraperitoneally (i.p.) injected 0.1 mg/kg of LPS in naive mice and, 1 h later, intravenously (i.v.) administered one 5 mg/kg dose of mTORi-nanobiologics (Figure 4A). Naive mice were used as negative controls, while LPS-injected PBS-treated animals served as placebo controls. We then applied a combination of *in vivo* ^18^F-FDG positron emission tomography (^18^F-FDG PET) imaging^43^^,^^44^^,^^45^ (Figure 4B), *ex vivo* gamma counting, and flow cytometric analyses. Compared to naive mice, we observed a 4-fold increase *ex vivo* in ^18^F-FDG bone marrow uptake following i.p. LPS injection, indicative of systemic hyperinflammation (Figure 4C, *p* < 0.0001). In the same analysis, LPS-injected mice that received the mTORi-nanobiologic treatment displayed significantly less bone marrow activity after 24 h (Figure 4C, *p* = 0.0007). Interestingly, no statistical significance between negative control and mTORi-nanobiologic treated groups was attained (*p* = 0.3967), suggestive of inflammation reduction to baseline levels. A similar trend was observed by *in vivo* standardized uptake value (SUV) analysis; although statistical significance was not attained (Figure S4A), most likely attributed to the lower sensitivity of PET scanners due to partial volume effects, background noise and limitations in spatial resolution.^46^^,^^47^

We set out to investigate these changes at the cellular level by flow cytometry analysis of bone marrow cells. We observed a reduction of Ly6C^hi^ monocytes after mTORi-nanobiologics administration, when compared to placebo-treated animals (Figure 4D, *p* = 0.0011). Similarly, neutrophil populations also underwent a 5-fold reduction after mTORi-nanobiologics treatment, when compared to the placebo group. However, those differences did not reach statistical significance (Figure 4E, *p* = 0.4133). Interestingly, a different trend was observed in the blood with decreased Ly6C^hi^ monocyte and neutrophil populations in animals of the placebo group (Figure S4B), despite the lack of statistical significance (*p* = 0.3624 and *p* = 0.5288). This phenomenon is most likely due to enhanced margination and cell migration to inflamed organs after LPS injection.^48^^,^^49^ However, mTORi-nanobiologics treatment restored Ly6C^hi^ monocyte and neutrophil levels to baseline (*p* = 0.2060 and *p* = 0.9707, respectively).

### mTORi-nanobiologics modulate ARDS-driven inflammation in mice

**Figure 5:**
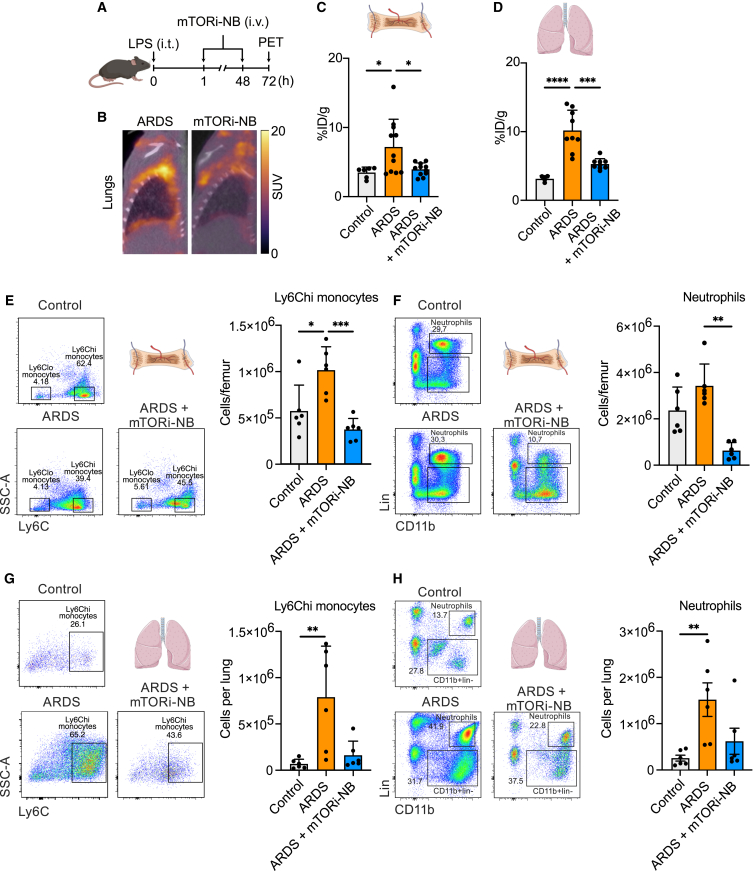
mTORi-nanobiologics reduce inflammation in ARDS mouse model (A) Schematic overview of the LPS-induced ARDS mouse model. (B) Representative fused ^18^F-FDG PET/CT images of the lungs of untreated (placebo control, left panel) and mTORi-nanobiologics-treated (right panel) animals. (C) *Ex vivo* quantification of bone marrow ^18^F-FDG uptake (*n* = 6–11). (D) *Ex vivo* quantification of ^18^F-FDG lung uptake (*n* = 4–9). (E) Representative flow cytometry plots showing Ly6C^hi^ monocytes in the bone marrow of control naive mice (negative control), untreated ARDS mice (placebo control), and ARDS mice treated with mTORi-nanobiologics, gated on live CD45^+^CD11b^+^lin^−^CD11c^−^ cells (left panel) and the associated quantification of bone marrow Ly-6C^hi^ monocytes (right panel, *n* = 6). (F) Representative flow cytometry plots showing neutrophils in the bone marrow of control naive mice (negative control), untreated ARDS mice (placebo control), and ARDS mice treated with mTORi-nanobiologics, gated on live CD45^+^ cells (left panel). The associated quantification of bone marrow neutrophils is also shown (right panel, *n* = 6). (G) Representative flow cytometry plots showing Ly6C^hi^ monocytes in the lungs of naive mice (negative control), untreated ARDS mice (placebo control), and ARDS mice treated with mTORi-nanobiologics, gated on live CD45^+^CD11b^+^lin^−^CD11c^−^ cells (left panel) and the associated quantification of lung Ly-6C^hi^ monocytes are also shown (right panel, *n* = 6). (H) Representative flow cytometry plots showing neutrophils in the lungs of control naive mice (negative control), untreated ARDS mice (placebo control), and ARDS mice treated with mTORi-nanobiologics, gated on live CD45^+^ cells (left panel). The associated quantification of lung neutrophils is also shown (right panel, *n* = 6). ARDS = placebo control, SUV = standardized uptake value. Data in (C)–(H) are plotted as mean ± SD. Statistical analyses were performed using the Shapiro-Wilk test, followed by one-way ANOVA (with Tukey’s multiple comparisons test) or Kruskal-Wallis (followed by Dunn’s multiple comparison test) according to Gaussian distribution. ∗*p* < 0.05, ∗∗*p* < 0.01, ∗∗∗*p* < 0.001, ∗∗∗∗*p* < 0.0001. See also Figure S5.

Encouraged by the anti-inflammatory effects of mTORi-nanobiologic therapy in systemic inflammation, we set out to test its efficacy in a mouse model of ARDS. This model involves intratracheally administering LPS (i.t., 0.1 mg/kg, Figure 5A). mTORi-nanobiologics treatment (or PBS for the placebo group) was intravenously injected 1 h and 48 h after LPS exposure, while the negative control group did not receive LPS. Animals were then ^18^F-FDG-PET imaged (Figure 5B) and euthanized for *ex vivo* radiometric assays. *Ex vivo* readouts of bone marrow ^18^F-FDG uptake showed 2-fold increase in LPS-injected animals (Figure 5C, *p* = 0.0271), while LPS-injected mTORi-nanobiologics-treated mice had significantly reduced ^18^F-FDG uptake, when compared to placebo group (Figure 5C, *p* = 0.0207). However, similar to the hyperinflammation model, these differences did not reach statistical significance during *in vivo* SUV analysis (Figure S5A).

*Ex vivo*^18^F-FDG uptake in the lungs at the same time point showed a 3-fold increase after ARDS induction (Figure 5D, *p* < 0.0001), when compared to naive control. Similar to the treatment response in the systemic hyperinflammation mouse model, we found that two i.v. doses of mTORi-nanobiologics (5 mg/kg) significantly reduced ^18^F-FDG uptake in the lungs (Figure 5D, *p* = 0.0001) to baseline (negative control) levels (*p* = 0.1940). These data were corroborated by *in vivo* SUV analysis (Figure S5B, *p* = 0.0024, 0.0379, and > 0.9999, respectively).

On a cellular level, mTORi-nanobiologics-treated animals also showed a significant reduction in both bone marrow Ly6C^hi^ monocytes and neutrophils, when compared to the placebo group (Figures 5E and 5F, *p* = 0.0006 and 0.0011, respectively). Similar trends were observed in the lungs (Figures 5G and 5H, *p* = 0.1168 and 0.1168, respectively) and in the neutrophils in the blood (Figure S5C, *p* = 0.0401).

## Discussion

Over the course of the past century, infection-related death rates have declined dramatically due to advancements in hygiene, the improved quality of life, and modern medicine.^50^ Nevertheless, sepsis induced by infection remains one of the most notorious killers worldwide.^2^^,^^51^^,^^52^ While sepsis is an immune-mediated condition, immunotherapy trials demonstrating clinical benefits are scarce, which has stagnated their development.^53^^,^^54^ However, the COVID-19 pandemic forced the medical community and pharma to refocus their attention on treating sepsis and hyperinflammation. Numerous trials were performed with existing drugs,^55^ such as anakinra^56^^,^^57^ and T cell targeting therapies.^58^ Although success rates varied considerably, it has become evident that with proper patient stratification immunotherapy has significant potential for treating infection-related complications, including sepsis.

In the current study, we found differential expression of genes related to the mTOR pathway in myeloid cells from COVID-19 patients. The link between mTOR and COVID-19-driven hyperinflammation has been investigated in previous studies.^59^^,^^60^ Basile et al. outlined that SARS-CoV-2 infection disrupts mTOR signaling and suggested that its inhibition could be a powerful tool for immune response regulation in COVID-19, a conclusion supported by Abu-Eid et al*.*^61^^,^^62^ Moreover, the correlation between COVID-19 immune dysregulation and mTOR effectors is further aggravated in subjects with underlying comorbidities such as diabetes^63^ and obesity.^64^

Indeed, our scRNA-Seq results indicate that SARS-CoV-2 infection upregulates mTOR-related genes involved in actin polymerization and cytoskeletal rearrangements, mechanisms that enhance immune cell trafficking and motility.^34^^,^^35^^,^^36^ These processes are key drivers of tissue infiltration and damage, often culminating in hyperinflammation.^65^^,^^66^^,^^67^ Furthermore, these genes also modulate PI-3 kinase signaling and, consequently, the production of pro-inflammatory cytokines and chemokines.^68^^,^^69^ In contrast, other mTOR downstream targets, such as Rps6, a component of the small (40S) ribosomal unit, were downregulated. This is also aligned with typical physiological responses to infection and could be part of a well-established feedback loop mechanism to counteract inflammation.^70^^,^^71^ In fact, evidence suggests that the activation of mTOR signaling concomitantly triggers compensatory processes to help restore metabolic and inflammatory homeostasis by downregulating downstream genes via insulin/IGF receptor and other tyrosine kinase receptors.^72^^,^^73^ However, these modulatory effects are often insufficient to counteract an exacerbated immune response, especially in hyperinflammation and can paradoxically further compromise patient health under certain conditions.^74^^,^^75^^,^^76^ Interestingly, certain changes in mTOR-related gene expression persisted in monocytes, even after disease recovery. Thus, we foresee that mTOR pathway regulation may be applied not only to mitigate direct consequences of SARS-CoV-2 infection, but also to reduce its chronic adverse effects^77^ and the aggravation of comorbidities, such as cardiovascular disease.^78^^,^^79^ Together, these findings highlight the complexity and dynamic nature of mTOR signaling, which combines pro-inflammatory and homeostasis restoration properties, and underscore its potential as a therapeutic target for addressing short and long-lasting effects of infection.

Drugs that inhibit mTOR, such as rapamycin, are Food and Drug Administration (FDA) approved for organ transplant rejection prevention, treatment of certain tumors,^80^ pulmonary lymphangioleiomyomatosis,^81^ and are applied in drug-eluding stents to prevent restenosis of coronary arteries.^82^ In addition, rapamycin has been studied for the treatment of lung injuries due to infection, including in SARS-CoV-2.^83^^,^^84^ However, the use of this medication for inhibition of hyperinflammation and its clinical manifestations (such as ARDS) has limitations, due to its immunosuppressive effects. It is well documented that rapamycin negatively impacts T cell function^28^ and consequently hampers immune responses to infectious agents. mTOR inhibition also leads to suppression of B cell development and proliferation, hampering the production of antigen-specific memory B cells.^85^ Moreover, despite its immunosuppressive function, impaired immune responses due to mTOR inhibition may also lead to unexpected pro-inflammatory effects. This paradoxical outcome is caused by decreased anti-inflammatory IL-10 production with parallel increase in expression and release of pro-inflammatory cytokine IL-12.^86^^,^^87^ A recent study also points out to the deleterious effects that rapamycin may have in non-immune tissues, such as the skeletal muscle, especially in older patients.^88^ Therefore, alternative strategies for more precise and cell-specific mTOR inhibition, which do not mute essential immune responses, must be engineered.

We previously introduced a nanobiologic platform for myeloid cell-specific immunoregulation. Nanobiologics are nanomedicines based on natural lipoprotein nanoparticles that integrate small molecule drugs.^89^ Owing to their lipoprotein features, nanobiologics are highly biocompatible, safe in large animals,^90^^,^^91^ and specifically target myeloid cells and their bone marrow progenitors. In a series of separate studies, we applied nanobiologics that are functionalized with rapamycin-based drugs for myeloid cell-specific mTOR regulation. We showed compelling therapeutic benefits of mTORi-nanobiologic treatment in mouse models of atherosclerosis^92^ and organ transplantation,^93^ without causing widespread immunosuppression. In the latter, mTORi-nanobiologics’ cellular specificity toward myeloid cells was characterized by flow cytometry. Important to clinical translation, mTORi-nanobiologics’ intravenous application is safe in non-human primates.^29^ Thus, we sought out to investigate the use of this mTORi-nanobiologics formulation in the context of infection-driven dysregulated immune response.

Our *in vitro* experiments on primary cells and mouse models uncovered mTORi-nanobiologic therapy’s compelling potential. In primary human immune cells, treatment prevented inflammation and trained immunity induction. Furthermore, our *in vivo* data provided new insights into the poorly understood temporal-spatial dynamics of mTOR inhibition across distinct models of inflammation. Hyperinflammation induced by intraperitoneal injection with LPS is a well-established model for acute systemic inflammation.^94^^,^^95^ In the 24-h post administration, LPS induced production of Ly6C^hi^ monocytes and neutrophils by the bone marrow, which migrated to inflamed organs, as evidenced by rapid depletion of circulating cells. This is in line with the bone marrow’s high metabolic activity measured by ^18^F-FDG. Conversely, intravenous mTORi-nanobiologic treatment evoked rapamycin-type effects on the bone marrow, hampering the development of Ly6C^hi^ monocytes and neutrophils,^96^^,^^97^ and impairing immune cells’ migratory capacity.^98^ Therefore, we observed elevated myeloid cell blood levels, despite their numbers being reduced in the bone marrow. Similarly, in the ARDS experiment LPS led to increased production of immune cells. However, the inflammation kinetics in ARDS are vastly different. Indeed, 72-h post LPS injection we observed persistent recruitment of immune cells to the lungs, which likely reflects replenishment of bone marrow cells after emergency myelopoiesis, as well as sustained influx to the bloodstream and affected organ. This difference was also observed in the treatment groups, where cell numbers for mTORi-nanobiologics-treated animals are stabilized to naive levels.

While our study provides clear proof-of-concept for myeloid cell-specific mTOR inhibition in infection-associated inflammation, it is limited to studying myeloid cells from COVID-19 patients only. However, mTOR dysregulation in myeloid cells is found in diverse infections, ranging from HIV^99^^,^^100^^,^^101^ to dengue.^102^^,^^103^ Besides viral infections, mTOR pathway involvement is also implicated in conditions, such as bacterial pneumonia^104^ and tuberculosis,^105^ providing much broader opportunities for our therapeutic approach.

The experimental models used in this study accurately reflect critical features of sepsis but have their limitations. We evaluated mTORi-nanobiologics in an *in vitro* model that involves primary cells from healthy subjects, which are exposed to inactivated SARS-CoV-2. We did not treat myeloid cells from COVID-19 patients with mTORi-nanobiologics. For the therapeutic studies in mouse models, we induced inflammation by administering LPS, rather than through actual infection. LPS is found in the outer membrane of gram-negative bacteria.^106^ It is a potent immune cell activator and is often used in well-established models of post-infection immune dysregulation.^107^^,^^108^^,^^109^ When administered intraperitoneally, LPS can trigger systemic hyperinflammation, whereas intratracheal instillation leads to ARDS-like symptoms. Both phenomena (hyperinflammation and ARDS) are common COVID-19 complications,^110^^,^^111^ hence overarching our *in vitro* data. We chose this approach because despite WT mice not being susceptible to SARS-CoV-2 infections due to differences in their ACE2 receptor structure,^112^ LPS still has high relevance in the context of infection-driven inflammation. With this strategy, our human data are integrated with our mouse data in a complementary fashion. However, future studies with diverse infection mouse models are required to optimize the treatment regimen and evaluate mTORi-nanobiologic therapy’s full potential in sepsis-associated inflammation.

In summary, our data demonstrate that mTOR dysregulation in the myeloid compartment is a compelling therapeutic target. While monocytes and neutrophils are essential for host defense, exacerbated release, activation, and accumulation of myeloid cells at the site of infection—and systemically—can be harmful and lead to sepsis and death. Our mTORi-nanobiologic treatment downregulates bone marrow activity in LPS-induced systemic inflammation and ARDS, thereby limiting the deleterious accumulation of these myeloid cells in vital organs, without widespread immunosuppression.

### Limitations of the study

Our study investigates the potential of mTORi-nanobiologic treatment for regulating post-infection hyperinflammation. However, we evaluated the efficacy of this therapy *in vivo* only in animal models. In humans, we relied on data from scRNA-seq and *in vitro* approaches. Further studies are needed to understand the systemic effects of mTORi-nanobiologics in patients. Moreover, our analyses focused on COVID-19 patients, ARDS and hyperinflammation models. We believe nanobiologics could be applied to other diseases with similar mechanisms of inflammation dysregulation. Future research should explore the efficacy of our therapy in a broader range of diseases.

## Resource availability

### Lead contact

Further information and requests for resources and reagents should be directed to and will be fulfilled by the lead contact, Prof. Dr. Willem J.M. Mulder (Willem.Mulder@radboudumc.nl).

### Materials availability

Unique/stable reagents will be made available on request, but we may require a payment and/or a completed Materials Transfer Agreement if there is potential for commercial application.

### Data and code availability

• scRNA-seq data presented in this study have been deposited at the European Genome-phenome Archive (EGA). Accession numbers are listed in the key resources table.
• This paper does not report original code.
• Any additional information required to reanalyze the data reported in this work paper is available from the lead contact upon request.

## Acknowledgments

We thank Heiner Schaal for providing us with the heat-inactivated SARS-CoV-2 Wuhan-Hu-1 variant and Professor David Williams for providing us with (β-1,3-(D)-glucan. This work was funded by W.J.M.M's NIHNHLBI (R01 HL144072 and P01 HL131478), NIHNIAID (P01 AI168258), NWO Vici (91818622), ERC Advanced Grant TOLERANCE (101019807), Leducq Foundation CHECKPOINT ATHERO and Trained Therapeutix Discovery. R.v.d.M. was supported by a Dutch Research Council (NWO) Vidi grant (19681). E.J.G.-B. was supported by the Horizon 2020 European Grants ImmunoSep and RISCinCOVID and the Horizon Health grant EPIC-CROWN-2 (granted to the Hellenic Institute for the Study of Sepsis). SARS-CoV-2 research in the M.S. lab is further supported by NIH NIAID R01 AI160706 and R01 DK130425.

## Author contributions

W.J.M.M. conceived and supervised the study; W.J.M.M. and Y.C.T. designed and managed experiments; T.J.B., A.Z., Y.L., B.v.G., J.H.C.M.K, E.L., N.P.R., H.M.J., S.H.M.S., F.J.M.H., E.J.G.-B., M.M.M., M.S., R.v.d.M., R.D., L.A.B.J., R.E.T., Z.A.F., M.M.T.v.L., A.J.P.T., and M.G.N. were involved in the design of specific experiments; Y.C.T., J.M., J.M.-F., W.W., G.P., Y.v.E., B.P., J.D., T.A., T.J.B., E.E.S.B., M.D.A., A.Z., S.A.S., Z.Z., W.L., Y.L., A.M., J.C.F., G.A.O.C., E.K., G.S., L.C. and A.J.P.T. performed experiments and data acquisition; Y.C.T., J.M., J.M.-F., T.J.B., E.E.S.B., A.Z., Z.Z., W.L., Y.L., E.K., M.O., G.S., Z.A.F., M.M.T.v.L., A.J.P.T., and W.J.M.M. performed analyses and interpretation of the data; Y.C.T. and W.J.M.M. wrote the manuscript; All authors provided input and approved the manuscript’s final content.

## Declaration of interests

W.J.M.M., L.A.B.J, M.N., and Z.A.F. are founders of Trained Therapeutix Discovery (TTxD). W.J.M.M is also a shareholder and CSO at the company. W.J.M.M. is founder, CTO and shareholder of BioTrip. Z.A.F. and W.J.M.M. are members of the board of TTxD. J.H.C.M.K., B.v.G., G.A.O.C., and T.J.B. are employees at TTxD. H.M.J. is a director at SyMO-Chem. S.H.M.S. and F.J.M.H. are employees at SyMO-Chem. M.S. has received unrelated research support from ArgenX N.V., Moderna, 7Hills, and Phio Pharmaceuticals. E.J.G.-B. has received honoraria from Abbott Products Operations, bioMérieux, GSK, UCB, Sobi AB and ThermoFisher Brahms GmbH, independent educational grants from Abbott Products Operations, AbbVie, bioMérieux Inc, Johnson & Johnson, InCyte, MSD, Novartis, UCB, Sanofi, and Sobi. M.M.M. is the scientific founder of Lemba Therapeutics. No other potential conflicts of interest relevant to this article exist.

## STAR★Methods

### Key resources table

**Table:** 

REAGENT or RESOURCE	SOURCE	IDENTIFIER
**Antibodies**		

CD90.2 (Thy-1.2) Monoclonal Antibody (53-2.1), eFluor™ 450	eBioScience	Cat#: 48-0902-82; RRID:AB_1272200
TER-119 Monoclonal Antibody (TER-119), eFluor™ 450	eBioScience	Cat#: 48-5821-82; RRID:AB_1518808
NK1.1 Monoclonal Antibody (PK136), eFluor™ 450	eBioScience	Cat#: 48-5941-82; RRID:AB_2043877
CD49b (Integrin alpha 2) Monoclonal Antibody (DX5), eFluor™ 450	eBioScience	Cat#: 48-5971-82; RRID:AB_10671541
CD45R (B220) Monoclonal Antibody (RA3-6B2), eFluor™ 450	eBioScience	Cat#: 48-0452-82; RRID:AB_1548761
Ly-6G Monoclonal Antibody (1A8-Ly6g), eFluor™ 450	eBioScience	Cat#: 48-9668-82; RRID:AB_2637124
Brilliant Violet 510(TM) anti-mouse CD45	BioLegend	Cat#: 101208; RRID:AB_2563061
Rat Anti-Ly-6C Monoclonal Antibody, FITC Conjugated, Clone AL-21	BD Biosciences	Cat#: 553104; RRID:AB_394628
PE anti-mouse/human CD11b	BioLegend	Cat#: 101208; RRID:AB_312791
APC anti-mouse CD11c	BioLegend	Cat# 117310; RRID:AB_313778
PE/Cyanine7 anti-mouse F4/8	BioLegend	Cat#: 123114; RRID:AB_893478
Rat Anti-CD16 / CD32 Monoclonal Antibody, Unconjugated, Clone 2.4G2	BD Biosciences	Cat#: 553142; RRID:AB_394657

**Bacterial and virus strains**		

SARS-CoV-2, Wuhan-Hu-1 variant (NRW-42 isolate)	Gift	N/A

**Biological samples**		

Donor blood	Homo sapiens	Ethical Committee Arnhem-Nijmegen (NL-nummer: NL32357.091.10)
Mouse samples	Mus musculus	Institutional Animal Care and Use Committees at the Icahn School of Medicine at Mount Sinai (LA12-00111)

**Chemicals, peptides, and recombinant proteins**		

1-palmitoyl-2-oleoyl-glycero-3-phosphocholine (POPC)	Avanti Polar Lipids	Cat#: 792453C-25mg; CAS: 26853-31-6
1-palmitoyl-2-hydroxy-sn-glycero-3-phosphocholine (PHPC)	Avanti Polar Lipids	CAS: 17364-16-8
Propane-1,2,3-triyl trioctanoate	Millipore Sigma	Cat#: T1978000; CAS: 538-23-8
Cholesterol	Avanti Polar Lipids	CAS: 57-88-5
Trichloromethane	Sigma-Aldrich	CAS: 67-66-3
Ethanenitrile	Sigma-Aldrich	CAS: 75-05-8
Methanol	Sigma-Aldrich	CAS: 67-56-1
Rapamycin	MCE	CAS: 53123-88-9
Ethenyl octadecanoate	Sigma-Aldrich	CAS: 111-63-7
Lipase	Sigma-Aldrich	CAS: 9001-62-1
Lipopolysaccharide from *Escherichia coli*	Sigma-Aldrich	Cat#: L2880
Percoll®	Sigma-Aldrich	Cat#: P1644
RBC Lysis Buffer	BioLegend	Cat#: 420301
DNase I from bovine pancreas	Sigma-Aldrich	Cat#: 11284932001
Collagenase A from *Clostridium histolyticum*	Sigma-Aldrich	Cat#: 10103586001
b-glucan (b-1,3-(D)-glucan)	Gift	N/A

**Critical commercial assays**		

Human IL-6 ELISA	R&D systems	Cat#: DY206
Human TNFα ELISA	R&D systems	Cat#: DY210
Human CXCL-9 ELISA	R&D systems	Cat#: DY392
Human CXCL-10 ELISA	R&D systems	Cat#: DY266
Human IL-8 ELISA	R&D systems	Cat#: DY208
Ficoll-Paque	GE Healthcare	Cat#: 17-1440-03
Roswell Park Memorial Institute medium (RPMI)	Invitrogen	Cat#: 22406031

**Deposited data**		

scRNA-seq raw data	Liu et al.^34^	EGA: S00001005529
scRNA-seq raw data	Schulte-Schrepping et al.^31^	EGA: S00001004571

**Experimental models: Organisms/strains**		

C57BL/6J	Jackson Laboratories	Cat#: 000664; RRID:IMSR_JAX:000664

**Software and algorithms**		

FACS DIVA Software	BD	In house license
FlowJo Software (v10.0.7)	TreeStar	https://www.flowjo.com/
GraphPad Prism (v10.2)	GraphPad Software	https://www.graphpad.com/
OsiriX (v11.0)	The Osirix Foundation	https://www.osirix-viewer.com/osirix/overview/

**Other**		

Carbon Film Supported Copper Square Mesh, size 200 mesh	Sigma-Aldrich	TEM-CF200CU

### Experimental model and study participant details

#### Mouse models of hyperinflammation and ARDS

All animal procedures were reviewed and approved by the Institutional Animal Care and Use Committees at the Icahn School of Medicine at Mount Sinai (LA12-00111), in accordance with federal regulations and the guidelines contained in the National Research Council Guide for the Care and Use of Laboratory Animals. Female C57BL/6 mice were purchased from the Jackson Laboratory at 7 to 10 weeks of age and randomly assigned to the different experimental groups. Animals were housed in a specific pathogen-free facility, maintained at constant room temperature of 25 ± 2°C. In the hyperinflammation model, mice received an intraperitoneal injection with LPS (0.1 mg/kg; E. coli O55:B5) 1 hour prior to intravenous injection with placebo (PBS, n=10) or mTORi-nanobiologics (5 mg/kg, n=11). Control animals (n=7) did not receive LPS injection. Similarly, for the ARDS model, animals were subjected to one intratracheal injection of LPS at 0.1 mg/kg, followed by two doses of PBS (n=10) or mTORi-nanobiologics (5 mg/kg, n=11) at 1 and 48 hours post LPS injection. Control group (n=6) did not receive LPS injection. Animals underwent ^18^F-FDG PET and *ex vivo* radiometric assays. In addition, 6 animals in each model group underwent flow cytometric analyses. A total of 2 lung samples per group were excluded from the ARDS *ex vivo* radiometric analyses due to PBS infiltration during perfusion, which interferes with the weight of the tissue, compromising the %ID/g calculation. In addition, 1 mouse was excluded from the *in vivo* ARDS analysis due to image reconstruction issues. In the placebo group of the cytokine storm analysis, 1 animal had to be euthanized before data collection due to LPS-induced complications.

#### Human monocyte isolation

Blood was collected from 12 healthy adult donors (n = 6 per batch) after written informed consent, according to the approval of the Ethical Committee Arnhem-Nijmegen (NL-nummer: NL32357.091.10) in compliance with the Medical Research Involving Human Subjects Act (WMO) and in accordance with the Declaration of Helsinki. Batch one included the analysis of IL-6 and TNF-α production. Later, a second batch of donors was recruited to perform additional analyses (CXCL-9, CXCL-10 and IL-8). However, in both batches, samples from each subject were subjected to all experimental conditions. Specifically, the same blood sample was exposed to different mTORi-nanobiologics concentrations, stimuli and control conditions. This within-subject set up minimizes individual variability. Information on age and sex of such donors are available on Table S3. PBMCs isolation was performed by differential density centrifugation.^113^ First, buffy coat was diluted in 200 mL of RPMI medium and overlayed in Ficoll (2:1). Samples were then centrifuged at 950 g for 15 minutes. PBMCs were collected from the interface between Ficoll and plasma-medium layers, washed with RPMI medium and centrifuged to 350g for 7 minutes. Supernatant was allowed to decant, and cells were resuspended in RPMI medium for one more round of washing and centrifugation. PBMCs suspension was brought to a 150-200 x 10^6^ cells/3 mL concentration and were added to 10 mL of hyperosmotic Percoll solution (4.85 mL of Percoll, 4.15 mL of sterile water and 1 mL of 1.6 M NaCl). Samples were then centrifuged at 580 g for 15 minutes at room temperature. Interface layer was collected, and cold PBS was added to a final volume of 50 mL. Samples were centrifuged at 350 g for 7 minutes at 4^o^C. Supernatant was removed, percoll-isolated monocytes (1 x 10^5^ cells) were re-suspended in RPMI culture medium and *in vitro* trained immunity experiments were performed.

### Method details

#### scRNA-seq of COVID patients

To understand the role of mTOR in COVID-19 patients, genes involved in mTOR signaling pathways were downloaded from KEGG (entry hsa04150) and analyzed in three independent scRNA-seq datasets including COVID-19 patients and healthy controls,^31^^,^^32^^,^^33^ with a total n = 157. Mild and severe patients were classified in accordance with the WHO clinical ordinal scale. The Seurat objects were imported and analyzed by Seurat package (version 4. 2.0) in R (version 4.2.0). In this study, we focus exclusively on genes related to the mTOR signaling pathway for the subsequent analysis.

#### Trained immunity assay

Monocytes were incubated with RPMI culture medium (RPMI medium Dutch modified), supplemented with 50 μg/mL gentamicin, 2 mM Glutamax, and 1 mM 2-oxopropanoate and stimulated with 1 μg/mL β-glucan (β-1,3-(D)-glucan, kindly provided by Professor David Williams) or heat-inactivated SARS-CoV-2 Wuhan-Hu-1 variant^38^ (8.9 x 10^4^ TCID50/mLl; NRW-42 isolate, kindly provided by Heiner Schaal, University Hospital Duesseldorf, Germany) or remained unstimulated for 24 h in the presence or absence of 1 or 10 μM mTORi-nanobiologics. Cells were washed with warm PBS and incubated for 5 days in culture medium supplemented with 10% human pool serum and medium was refreshed once. Cells were restimulated with 10 ng/mL *E. coli* LPS. After 24 h, supernatants were collected and stored at -20°C until analysis. Cytokine production from human cells was determined in supernatants using commercial ELISA kits for IL-6, TNF-α, CXCL-9, CXCL-10 and IL-8, following manufacturer’s instructions.

#### Preparation and characterization of mTOR inhibitor-loaded nanobiologics

Trained Therapeutix Discovery (Oss, the Netherlands) provided one of their proprietary rapamycin lipophilic mTOR inhibitor prodrugs. Stock solutions (10 mg/mL) in trichloromethane of POPC (250 μL), PHPC (65 μL), cholesterol (35 μL), propane-1,2,3-triyl trioctanoate (1000 μL) and rapamycin prodrug (200 μL) were combined in a 20 mL vial and dried under vacuum to yield a lipid film. The film was then dissolved in 3 mL ethanenitrile /methanol mixture (95:5 vol. %) and sonicated using an ultrasonic bath for 5 minutes. Separately, 24 mL PBS solution at 0.1 mg/mL ApoA-1 protein was prepared. Microfluidic setup with herringbone mixer was employed, loading both solutions simultaneously with a flow rate of 0.75 mL/min and 6 mL/min, for lipid solution and Apo-A1 solution, respectively. The nanoparticle suspension was concentrated to approximately 5 mL by centrifugal filtration (100k MWCO Vivaspin tube at 4000 rpm), followed by the addition of 10 mL of PBS. This step was repeated twice ensuring the removal of free components from the solution. The washed solution, concentrated to approximately 3 mL, was filtered using 0.22 μm PES syringe filter, resulting in the final mTORi-nanobiologics.

#### Nanoparticle quality control and characterization

Dynamic light scattering measurements of the nanotherapeutics were performed on a Malvern Zetasizer Ultra, indicating a mean size of 50 nm (based on number distribution) and a dispersity of 0.1 – 0.2, in line with our previous results.^29^ The rapamycin prodrug concentration was determined in triplicate by HPLC using a photodiode array detector (λ = 278 nm), diluting the nanobiologics in acetonitrile (1:50). Typical drug concentration were 1.5 mg/mL and drug incorporation efficiencies > 70%. For the cryogenic electron microscopy assay, the surface of 200-mesh lacey carbon supported copper grids was plasma charged for 40 seconds using a carbon coater (Cressington 208). Subsequently, 3 μl of nanobiologics sample (∼ 1 mg protein/mLl) was pipetted on a grid and vitrified into a thin film by plunge vitrification in liquid ethane. This step was performed by using an automated robot (FEI Vitrobot Mark IV). Cryo-EM imaging was acquired on the cryo-transmission electron microscope TITAN (Thermo Fisher), equipped with a field emission gun (FEG), a post-column Gatan imaging filter (model 2002) and a post-GIF 2k × 2k Gatan CCD camera (model 794). The imaging was performed at 300 kV acceleration voltage in bright-field TEM mode with zero-loss energy filtering at 24,000× magnification (dose rate of 11.8 e-/Å2·s), and 1s acquisition time.

#### *In vivo* PET/CT imaging and *ex vivo* measurements of radioactivity in mice

Static PET imaging was performed on mice at 24 (cytokine storm model) or 72 hours (ARDS model) after LPS administration. Animals were first fasted for 16 hours and then injected with ^18^F-FDG (∼12.84 ± 2.52 MBq) via their lateral tail vein and kept under 1% isoflurane anesthesia for 60 minutes to allow for tracer circulation. Anesthesia was maintained while animals were placed in the PET scanner (Mediso nanoScan PET/CT). First, a whole-body CT scan was performed (energy, 50 kVp; current, 180 μAs; isotropic voxel size, 0.25 mm) followed by a PET acquisition time of 20 minutes. Reconstruction was performed with attenuation correction using the TeraTomo 3D reconstruction algorithm from the Mediso Nucline software. The coincidences were filtered with an energy window between 400 and 600 keV. The voxel size was isotropic with 0.4-mm width, and the reconstruction was applied for four full iterations, six subsets per iteration. Immediately after the PET/CT scan, animals were euthanized for *ex vivo* radioactivity concentration measurements using a Wizard^2^ 2480 automatic gamma counter by PerkinElmer, which reported as a decay corrected percentage of injected dose per gram (%ID/g).

#### Image analysis

Upon reconstruction, image analysis was performed using Osirix MD, version 11.0. Whole-body CT images were fused with PET images and analyzed in a coronal plane. Regions of interest (ROIs) were drawn on organs of interest, using the femur for assessment of bone marrow uptake. Mean standardized uptake values (SUVs) were calculated for each ROI.

#### Flow cytometry of mouse samples

For the flow cytometry analysis, animals were euthanized and perfused with cold PBS. Tissues of interest were harvested and processed. Bone marrow was extracted from the femur and strained though a 70 μm filter. Samples were subsequently incubated with RBC lysis buffer for 30 seconds and washed with PBS. Blood samples were lysed using RBC lysis buffer for 4 minutes, followed by two additional rounds of lysis and washing with PBS. Lung samples were treated with enzymatic solution containing 1 mg/mL of DNAse I and 5 mg/mL of Collagenase A in PBS supplemented with 0.5% FBS for 50 minutes at 37°C before being passed through a 70 μm filter and washed with PBS. Samples were stained with antibodies against CD45, Ly6C, CD11b, CD11c and F4/80 and a lineage cocktail containing antibodies against CD90.2, TER-119, NK1.1, CD49b, CD45R and Ly6G, for 30 minutes on ice. DAPI was used as a viability stain. Counting beads were used for absolute quantification of leukocyte subsets. Data were acquired on LSR Fortessa (BD Biosciences) and analyzed using FlowJo v10.0.7 (Tree Star).

#### Figure design

Schematic figures were prepared using BioRender (BioRender.com) and Servier Medical Art (smart.servier.com).

### Quantification and statistical analysis

#### Statistics

All results are presented as mean with standard deviation (SD), unless otherwise stated. Details on n-numbers are disclosed in the figure legend. Statistical tests were performed using GraphPad Prism 10.2. *In vitro* data was analyzed using Wilcoxon signed-rank test. α < 0.05 represents statistical significance. For multiple comparisons in the mouse study, data was tested for normality using Shapiro-Wilk test. Subsequent Ordinary one-way ANOVA test was employed for normally distributed data, followed by Tukey’s multiple comparison test, while non-parametric Kruskal-Wallis test was applied to non-Gaussian distributed data, followed by Dunn’s multiple comparison test. ∗p < 0.05, ∗∗p < 0.01, ∗∗∗p < 0.001, ∗∗∗∗p < 0.0001. For the analysis of the scRNA-Seq data, in each cell type from each dataset, the differentially expressed genes between every two disease conditions were identified using FindMarkers() function by Wilcoxon rank sum test. In the Bonn and Berlin cohorts, Bonferroni correction was used to control false discovery rate for multiple test results. Specifically, in the MHH50 cohort, we used the raw p < 0.05 to define significant differentially expressed genes in the convalescent COVID-19 dataset regarding the subtle differences between convalescent patients and healthy control.

### Additional resources

Patient cells analyses used here were obtained from data collected in previous studies.^31^^,^^32^^,^^33^

In addition to the deposition of the raw sequencing data on EGA, we provide an interactive platform for data inspection and analysis via FASTGenomics. The FASTGenomics platform (fastgenomics.org) provides processed count tables of the datasets generated in this study as well as key analytical results, such as UMAP coordinates and cluster identities, and the code written to analyze the respective data.
