# Supplementary material for: Targeting mTOR in myeloid cells prevents infection-associated inflammation
Source: iScience. 2025 Mar 4;28(4):112163. doi: 10.1016/j.isci.2025.112163 (PMC11964677; doi:10.1016/j.isci.2025.112163)
Supplement: Document S1. Figures S1–S5 and Tables S1–S3 [file mmc1.pdf]

iScience, Volume *28*

Supplemental information

Targeting mTOR in myeloid cells

prevents infection-associated inflammation

Yohana C. Toner, Jazz Munitz, Geoffrey Prevot, Judit Morla-Folch, William Wang, Yuri van Elsas, Bram Priem, Jeroen Deckers, Tom Anbergen, Thijs J. Beldman, Eliane E.S. Brechbühl, Muhammed D. Aksu, Athanasios Ziogas, Sebastian A. Sarlea, Mumin Ozturk, Zhenhua Zhang, Wenchao Li, Yang Li, Alexander Maier, Jessica C. Fernandes, Glenn A.O. Cremers, Bas van Genabeek, Joost H.C.M. Kreijtz, Esther Lutgens, Niels P. Riksen, Henk M. Janssen, Serge H.M. Söntjens, Freek J.M. Hoeben, Ewelina Kluza, Gagandeep Singh, Evangelos J. Giamarellos-Bourboulis, Michael Schotsaert, Raphaël Duivenvoorden, Roy van der Meel, Leo A.B. Joosten, Lei Cai, Ryan E. Temel, Zahi A. Fayad, Musa M. Mhlanga, Mandy M.T. van Leent, Abraham J.P. Teunissen, Mihai G. Netea, and Willem J.M. Mulder

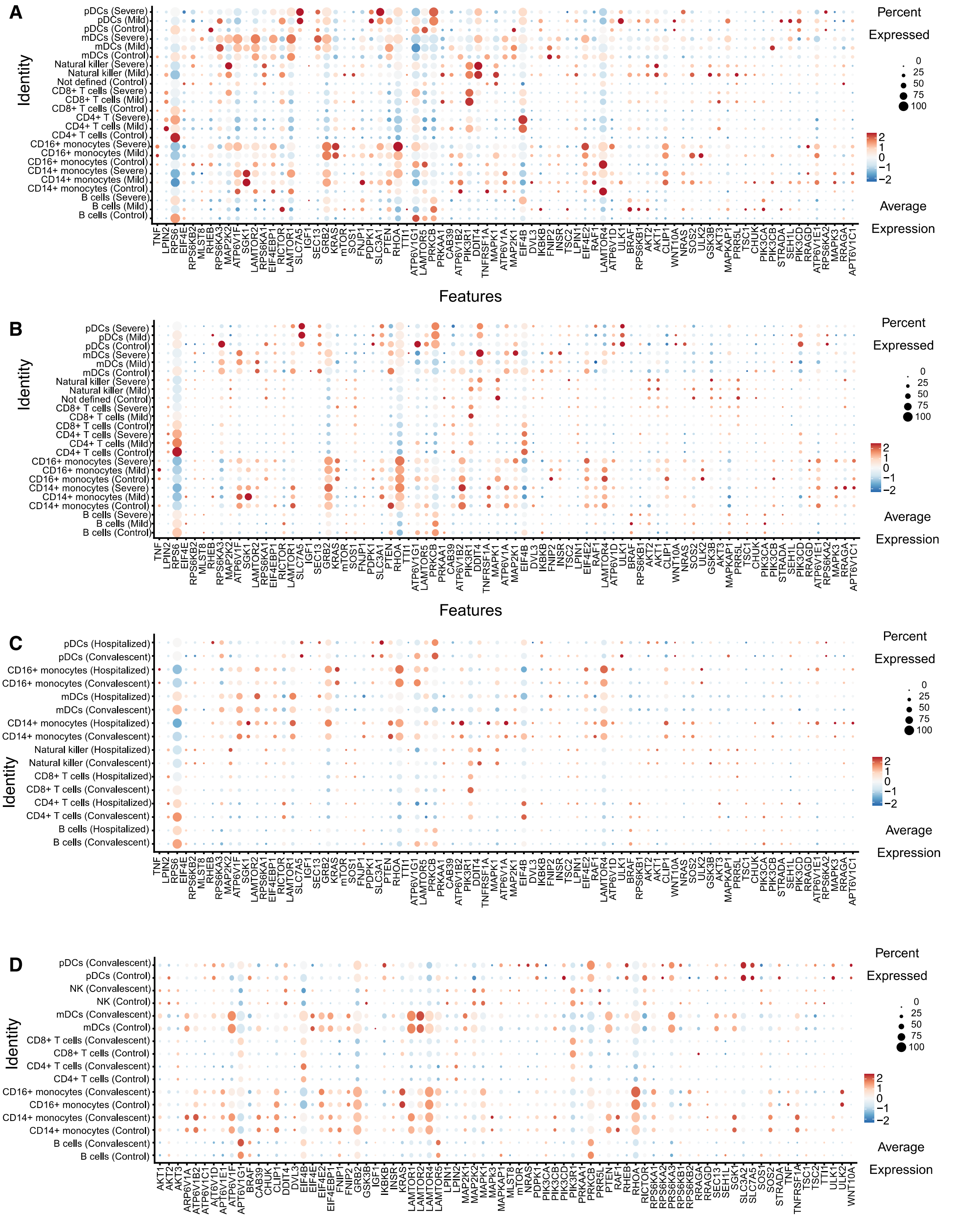

Supplemental Figure 1, related to Figure 1. mTOR signaling pathway genes expression profiles in different COVID-19 datasets. Expression profile of mTOR signaling pathway genes that are differentially expressed in at least one comparison in the Berlin (**A**), Bonn (**B**)[1], MHH50 (**C**)[2] or Convalescent COVID-19 (**D**)[3] cohorts. Differentially expressed genes between every two disease conditions were identified using FindMarkers() function by Wilcoxon rank sum test.

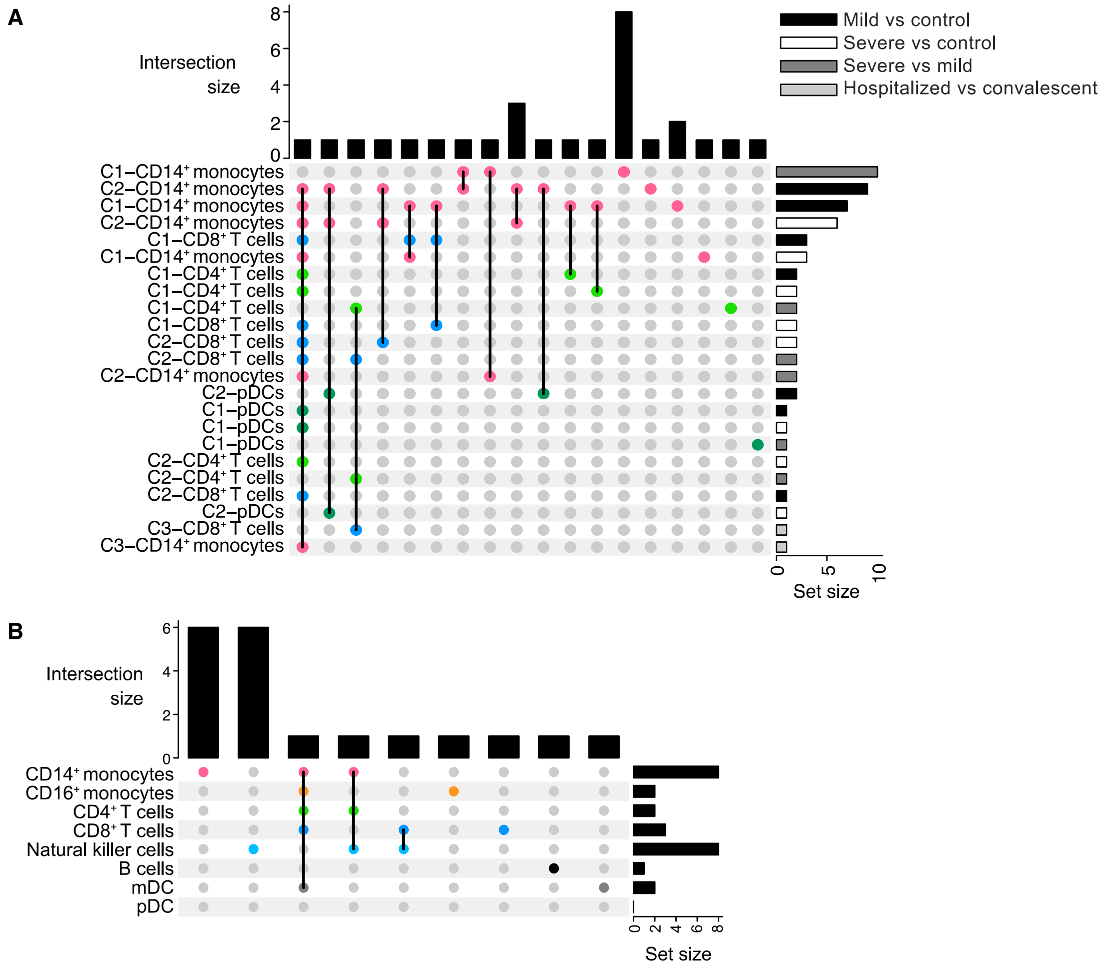

Supplemental Figure 2, related to Figure 1. mTOR signaling pathway downregulated gene expression profiles (**A**) Downregulated differentially expressed genes across the Berlin (C1), Bonn (C2)[1], and MHH50 (C3)[2] datasets. (**B**) The number of downregulated mTOR genes in specific cell types in convalescent COVID-19 patients[3] (C4). Differentially expressed genes between every two disease conditions were identified using FindMarkers() function by Wilcoxon rank sum test.

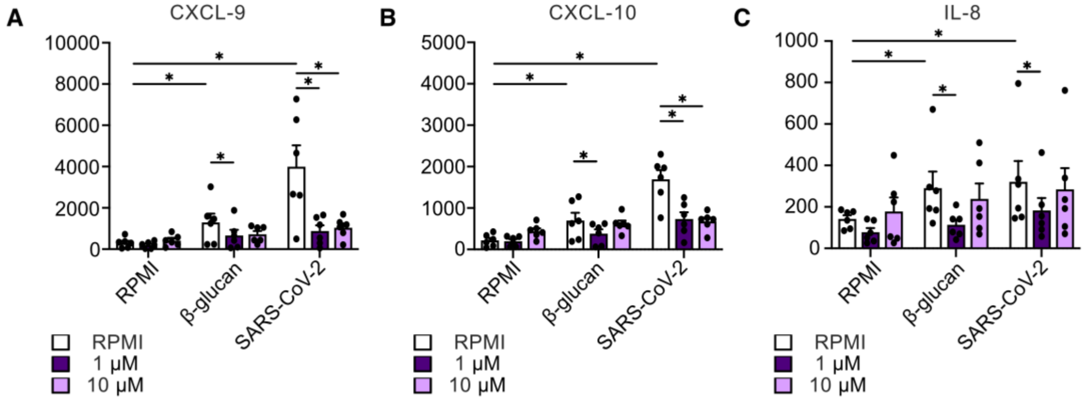

Supplemental Figure 3, Related to Figure 3. mTORi-nanobiologics reduces pro-inflammatory chemokine production in human monocytes *in vitro*. (**A**) *In vitro* trained immunity assay showing CXCL-9 production upon LPS restimulation. (**B**) *In vitro* trained immunity assay showing CXCL-10 production upon LPS restimulation. (**C**) *In vitro* trained immunity assay showing IL-8 production upon LPS restimulation. Statistical analysis was performed using the Wilcoxon signed-rank test. *p<0.05.

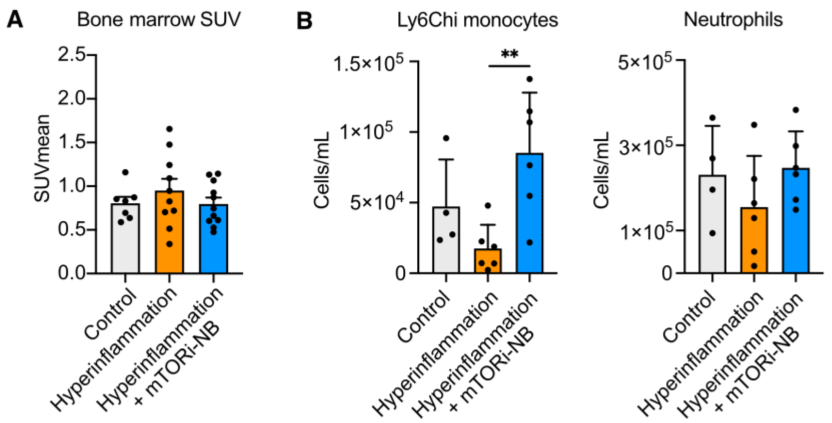

Supplemental Figure 4, related to Figure 4. *In vivo* assessment of organ inflammation in the hyperinflammation mouse model. (**A**) PET-based quantification of ^{18}F-FDG in the bone marrow 24 h post LPS injection (n = 7-11). (**B**) Quantification of blood Ly-6C^{hi} monocytes (left) and neutrophils (right, n=4-6). Hyperinflammation = placebo control, SUV = standardized uptake value. Statistical analyses were performed using the Shapiro-Wilk test, followed by one-way ANOVA (with Tukey's multiple comparisons test). **p<0.01.

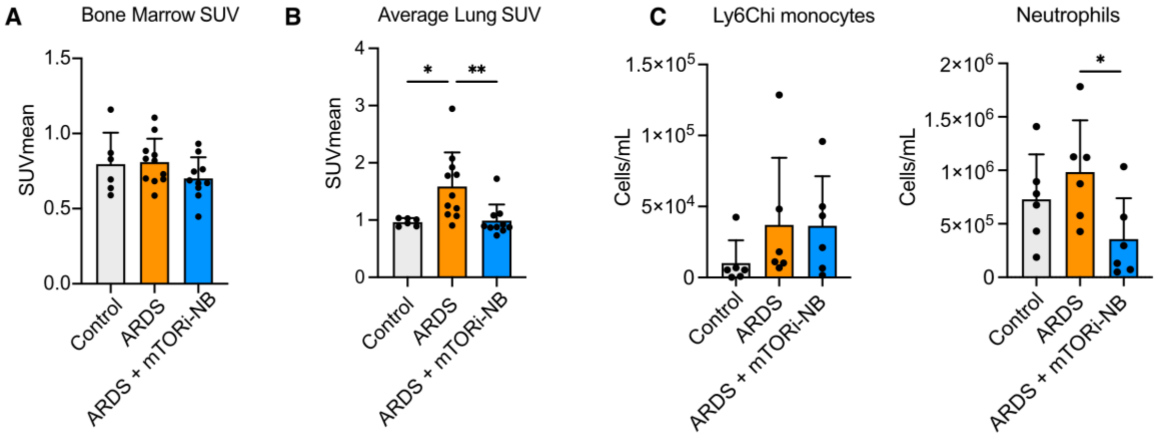

Supplemental Figure 5, related to Figure 5. *In vivo* assessment of organ inflammation in the ARDS mouse model. (**A**) PET-based quantification of ^{18}F-FDG in the bone marrow 72 h post LPS injection (n = 6-11). (**B**) PET-based quantification of ^{18}F-FDG in the lungs 72 h post LPS injection (n = 6-10). (**C**) Quantification of blood Ly-6C^{hi} monocytes (left) and neutrophils (right, n=6). SUV = standardized uptake value. Statistical analyses were performed using the Shapiro-Wilk test, followed by one-way ANOVA (with Tukey's multiple comparisons test) or Kruskal-Wallis (followed by Dunn's multiple comparison test) according to Gaussian distribution. *p<0.05, **p<0.01.

Supplementary Table 1, related to Figure 1. Genes involved in mTOR signaling pathway downloaded from KEGG (hsa04150)

KEGG Gene entry	Gene symbol	Description
8140	SLC7A5	solute carrier family 7 member 5 [KO:K13780]
6520	SLC3A2	solute carrier family 3 member 2 [KO:K06519]
153129	SLC38A9	solute carrier family 38 member 9 [KO:K14995]
523	ATP6V1A	ATPase H+ transporting V1 subunit A [KO:K02145] [EC:7.1.2.2]
525	ATP6V1B1	ATPase H+ transporting V1 subunit B1 [KO:K02147]
526	ATP6V1B2	ATPase H+ transporting V1 subunit B2 [KO:K02147]

245973	ATP6V1C2	ATPase H+ transporting V1 subunit C2 [KO:K02148]
528	ATP6V1C1	ATPase H+ transporting V1 subunit C1 [KO:K02148]
51382	ATP6V1D	ATPase H+ transporting V1 subunit D [KO:K02149]
90423	ATP6V1E2	ATPase H+ transporting V1 subunit E2 [KO:K02150]
529	ATP6V1E1	ATPase H+ transporting V1 subunit E1 [KO:K02150]
9296	ATP6V1F	ATPase H+ transporting V1 subunit F [KO:K02151]
9550	ATP6V1G1	ATPase H+ transporting V1 subunit G1 [KO:K02152]
127124	ATP6V1G3	ATPase H+ transporting V1 subunit G3 [KO:K02152]
534	ATP6V1G2	ATPase H+ transporting V1 subunit G2 [KO:K02152]
51606	ATP6V1H	ATPase H+ transporting V1 subunit H [KO:K02144]
55004	LAMTOR1	late endosomal/lysosomal adaptor, MAPK and MTOR activator 1 [KO:K20397]
28956	LAMTOR2	late endosomal/lysosomal adaptor, MAPK and MTOR activator 2 [KO:K20398]
8649	LAMTOR3	late endosomal/lysosomal adaptor, MAPK and MTOR activator 3 [KO:K04370]
389541	LAMTOR4	late endosomal/lysosomal adaptor, MAPK and MTOR activator 4 [KO:K20399]
10542	LAMTOR5	late endosomal/lysosomal adaptor, MAPK and MTOR activator 5 [KO:K16344]
201163	FLCN	folliculin [KO:K09594]
96459	FNIP1	folliculin interacting protein 1 [KO:K20400]
57600	FNIP2	folliculin interacting protein 2 [KO:K20401]
10670	RRAGA	Ras related GTP binding A [KO:K16185]
10325	RRAGB	Ras related GTP binding B [KO:K16185]
64121	RRAGC	Ras related GTP binding C [KO:K16186]
58528	RRAGD	Ras related GTP binding D [KO:K16186]
83667	SESN2	sestrin 2 [KO:K20394]
652968	CASTOR1	cytosolic arginine sensor for mTORC1 subunit 1 [KO:K23080]
729438	CASTOR2	cytosolic arginine sensor for mTORC1 subunit 2 [KO:K23081]
54468	MIOS	meiosis regulator for oocyte development [KO:K20407]
81929	SEH1L	SEH1 like nucleoporin [KO:K14299]
84219	WDR24	WD repeat domain 24 [KO:K20408]
79726	WDR59	WD repeat domain 59 [KO:K20409]
6396	SEC13	SEC13 homolog, nuclear pore and COPII coat complex component [KO:K14004]
9681	DEPDC5	DEP domain containing 5, GATOR1 subcomplex subunit [KO:K20404]
10641	NPRL2	NPR2 like, GATOR1 complex subunit [KO:K20405]
8131	NPRL3	NPR3 like, GATOR1 complex subunit [KO:K20406]
6502	SKP2	S-phase kinase associated protein 2 [KO:K03875]
220441	RNF152	ring finger protein 152 [KO:K15705] [EC:2.3.2.27]
57521	RPTOR	regulatory associated protein of MTOR complex 1 [KO:K07204]
84335	AKT1S1	AKT1 substrate 1 [KO:K16184]
2475	MTOR	mechanistic target of rapamycin kinase [KO:K07203] [EC:2.7.11.1]
64798	DEPTOR	DEP domain containing MTOR interacting protein [KO:K20402]
64223	MLST8	MTOR associated protein, LST8 homolog [KO:K08266]
9894	TELO2	telomere maintenance 2 [KO:K11137]
9675	TTI1	TELO2 interacting protein 1 [KO:K20403]
6249	CLIP1	CAP-Gly domain containing linker protein 1 [KO:K10421]
2887	GRB10	growth factor receptor bound protein 10 [KO:K20064]
23175	LPIN1	lipin 1 [KO:K15728] [EC:3.1.3.4]
64900	LPIN3	lipin 3 [KO:K15728] [EC:3.1.3.4]
9663	LPIN2	lipin 2 [KO:K15728] [EC:3.1.3.4]
8408	ULK1	unc-51 like autophagy activating kinase 1 [KO:K21357] [EC:2.7.11.1]
9706	ULK2	unc-51 like autophagy activating kinase 2 [KO:K08269] [EC:2.7.11.1]
1978	EIF4EBP1	eukaryotic translation initiation factor 4E binding protein 1 [KO:K07205]
1977	EIF4E	eukaryotic translation initiation factor 4E [KO:K03259]
9470	EIF4E2	eukaryotic translation initiation factor 4E family member 2 [KO:K03259]

253314	EIF4E1B	eukaryotic translation initiation factor 4E family member 1B [KO:K03259]
6198	RPS6KB1	ribosomal protein S6 kinase B1 [KO:K04688] [EC:2.7.11.1]
6199	RPS6KB2	ribosomal protein S6 kinase B2 [KO:K04688] [EC:2.7.11.1]
1975	EIF4B	eukaryotic translation initiation factor 4B [KO:K03258]
6194	RPS6	ribosomal protein S6 [KO:K02991]
92335	STRADA	STE20 related adaptor alpha [KO:K08271]
55437	STRADB	STE20 related adaptor beta [KO:K17532]
6794	STK11	serine/threonine kinase 11 [KO:K07298] [EC:2.7.11.1]
51719	CAB39	calcium binding protein 39 [KO:K08272]
81617	CAB39L	calcium binding protein 39 like [KO:K08272]
5562	PRKAA1	protein kinase AMP-activated catalytic subunit alpha 1 [KO:K07198] [EC:2.7.11.11]
5563	PRKAA2	protein kinase AMP-activated catalytic subunit alpha 2 [KO:K07198] [EC:2.7.11.11]
7248	TSC1	TSC complex subunit 1 [KO:K07206]
7249	TSC2	TSC complex subunit 2 [KO:K07207]
51256	TBC1D7	TBC1 domain family member 7 [KO:K20396]
107080638	TBC1D7-LOC100130357	readthrough [KO:K20396]
6009	RHEB	Ras homolog, mTORC1 binding [KO:K07208]
54541	DDIT4	DNA damage inducible transcript 4 [KO:K08270]
7471	WNT1	Wnt family member 1 [KO:K03209]
7472	WNT2	Wnt family member 2 [KO:K00182]
7482	WNT2B	Wnt family member 2B [KO:K00182]
7473	WNT3	Wnt family member 3 [KO:K00312]
89780	WNT3A	Wnt family member 3A [KO:K00312]
54361	WNT4	Wnt family member 4 [KO:K00408]
7474	WNT5A	Wnt family member 5A [KO:K00444]
81029	WNT5B	Wnt family member 5B [KO:K00444]
7475	WNT6	Wnt family member 6 [KO:K00445]
7476	WNT7A	Wnt family member 7A [KO:K00572]
7477	WNT7B	Wnt family member 7B [KO:K00572]
7478	WNT8A	Wnt family member 8A [KO:K00714]
7479	WNT8B	Wnt family member 8B [KO:K00714]
7483	WNT9A	Wnt family member 9A [KO:K01064]
7484	WNT9B	Wnt family member 9B [KO:K01064]
7480	WNT10B	Wnt family member 10B [KO:K01357]
80326	WNT10A	Wnt family member 10A [KO:K01357]
7481	WNT11	Wnt family member 11 [KO:K01384]
51384	WNT16	Wnt family member 16 [KO:K01558]
8321	FZD1	frizzled class receptor 1 [KO:K02432]
8324	FZD7	frizzled class receptor 7 [KO:K02432]
2535	FZD2	frizzled class receptor 2 [KO:K02235]
7976	FZD3	frizzled class receptor 3 [KO:K02329]
8322	FZD4	frizzled class receptor 4 [KO:K02354]
7855	FZD5	frizzled class receptor 5 [KO:K02375]
8325	FZD8	frizzled class receptor 8 [KO:K02375]
8323	FZD6	frizzled class receptor 6 [KO:K02376]
11211	FZD10	frizzled class receptor 10 [KO:K02842]
8326	FZD9	frizzled class receptor 9 [KO:K02842]
4041	LRP5	LDL receptor related protein 5 [KO:K03068]
4040	LRP6	LDL receptor related protein 6 [KO:K03068]
1857	DVL3	dishevelled segment polarity protein 3 [KO:K02353]
1856	DVL2	dishevelled segment polarity protein 2 [KO:K02353]
1855	DVL1	dishevelled segment polarity protein 1 [KO:K02353]
2932	GSK3B	glycogen synthase kinase 3 beta [KO:K03083] [EC:2.7.11.26]
7124	TNF	tumor necrosis factor [KO:K03156]
7132	TNFRSF1A	TNF receptor superfamily member 1A [KO:K03158]
3551	IKBKB	inhibitor of nuclear factor kappa B kinase subunit beta [KO:K07209] [EC:2.7.11.10]
3630	INS	insulin [KO:K04526]
3479	IGF1	insulin like growth factor 1 [KO:K05459]
3643	INSR	insulin receptor [KO:K04527] [EC:2.7.10.1]
3480	IGF1R	insulin like growth factor 1 receptor [KO:K05087] [EC:2.7.10.1]

2885	GRB2	growth factor receptor bound protein 2 [KO:K04364]
6654	SOS1	SOS Ras/Rac guanine nucleotide exchange factor 1 [KO:K03099]
6655	SOS2	SOS Ras/Rho guanine nucleotide exchange factor 2 [KO:K03099]
3265	HRAS	HRas proto-oncogene, GTPase [KO:K02833]
3845	KRAS	KRAS proto-oncogene, GTPase [KO:K07827]
4893	NRAS	NRAS proto-oncogene, GTPase [KO:K07828]
673	BRAF	B-Raf proto-oncogene, serine/threonine kinase [KO:K04365] [EC:2.7.11.1]
5894	RAF1	Raf-1 proto-oncogene, serine/threonine kinase [KO:K04366] [EC:2.7.11.1]
5604	MAP2K1	mitogen-activated protein kinase kinase 1 [KO:K04368] [EC:2.7.12.2]
5605	MAP2K2	mitogen-activated protein kinase kinase 2 [KO:K04369] [EC:2.7.12.2]
5594	MAPK1	mitogen-activated protein kinase 1 [KO:K04371] [EC:2.7.11.24]
5595	MAPK3	mitogen-activated protein kinase 3 [KO:K04371] [EC:2.7.11.24]
6197	RPS6KA3	ribosomal protein S6 kinase A3 [KO:K04373] [EC:2.7.11.1]
6195	RPS6KA1	ribosomal protein S6 kinase A1 [KO:K04373] [EC:2.7.11.1]
6196	RPS6KA2	ribosomal protein S6 kinase A2 [KO:K04373] [EC:2.7.11.1]
27330	RPS6KA6	ribosomal protein S6 kinase A6 [KO:K04373] [EC:2.7.11.1]
3667	IRS1	insulin receptor substrate 1 [KO:K16172]
5295	PIK3R1	phosphoinositide-3-kinase regulatory subunit 1 [KO:K02649]
5296	PIK3R2	phosphoinositide-3-kinase regulatory subunit 2 [KO:K02649]
8503	PIK3R3	phosphoinositide-3-kinase regulatory subunit 3 [KO:K02649]
5290	PIK3CA	phosphatidylinositol-4,5-bisphosphate 3-kinase catalytic subunit alpha [KO:K00922] [EC:2.7.1.153]
5293	PIK3CD	phosphatidylinositol-4,5-bisphosphate 3-kinase catalytic subunit delta [KO:K00922] [EC:2.7.1.153]
5291	PIK3CB	phosphatidylinositol-4,5-bisphosphate 3-kinase catalytic subunit beta [KO:K00922] [EC:2.7.1.153]
5728	PTEN	phosphatase and tensin homolog [KO:K01110] [EC:3.1.3.16 3.1.3.48 3.1.3.67]
5170	PDPK1	3-phosphoinositide dependent protein kinase 1 [KO:K06276] [EC:2.7.11.1]
207	AKT1	AKT serine/threonine kinase 1 [KO:K04456] [EC:2.7.11.1]
208	AKT2	AKT serine/threonine kinase 2 [KO:K04456] [EC:2.7.11.1]
10000	AKT3	AKT serine/threonine kinase 3 [KO:K04456] [EC:2.7.11.1]
1147	CHUK	component of inhibitor of nuclear factor kappa B kinase complex [KO:K04467] [EC:2.7.11.10]
79109	MAPKAP1	MAPK associated protein 1 [KO:K20410]
253260	RICTOR	RPTOR independent companion of MTOR complex 2 [KO:K08267]
55615	PRR5	proline rich 5 [KO:K20411]
79899	PRR5L	proline rich 5 like [KO:K20411]
387	RHOA	ras homolog family member A [KO:K04513]
5578	PRKCA	protein kinase C alpha [KO:K02677] [EC:2.7.11.13]
5579	PRKCB	protein kinase C beta [KO:K19662] [EC:2.7.11.13]
5582	PRKCG	protein kinase C gamma [KO:K19663] [EC:2.7.11.13]
6446	SGK1	serum/glucocorticoid regulated kinase 1 [KO:K13302] [EC:2.7.11.1]

Supplementary Table 2, related to Figure 1. Cohort details scRNA-Sequencing analysis

Cohort	Sex (M/F)	Age	Status
Bonn (C1)	10/6; 4/4; 4/6	57,6; 72,8; 70,4	16 control; 8 mild; 10 severe
Berlin (C2)	N/A; 6/2; 6;4	N/A; 56,5; 63,1	12 control; 8 mild; 10 severe
MHH50 (C3)	17/10; 25; 12	N/A	27 convalescent; 37 hospitalized
Convalescent (C4)	7/7; 7/8	60; 50	14 convalescent; 15 control

Supplementary Table 3, related to Figure 3. Cohort details primary human cells in vitro analysis

Batch	Sex (M/F)	Age
1	F	24
1	M	27
1	F	25
1	F	27
1	F	31
1	M	35
2	M	44
2	M	61
2	F	57
2	M	56
2	M	51
2	F	30

REFERENCES

1. Schulte-Schrepping, J., Reusch, N., Paclik, D., Baßler, K., Schlickeiser, S., Zhang, B., Krämer, B., Krammer, T., Brumhard, S., Bonaguro, L., et al. (2020). Severe COVID-19 Is Marked by a Dysregulated Myeloid Cell Compartment. Cell *182*, 1419-1440.e23. 10.1016/j.cell.2020.08.001.
2. Zhang, B., Zhang, Z., Koeken, V.A.C.M., Kumar, S., Aillaud, M., Tsay, H.-C., Liu, Z., Kraft, A.R.M., Soon, C.F., Odak, I., et al. (2022). Altered and allele-specific open chromatin landscape reveals epigenetic and genetic regulators of innate immunity in COVID-19. Cell genomics, 100232. 10.1016/j.xgen.2022.100232.
3. Liu, Z., Kilic, G., Li, W., Bulut, O., Gupta, M.K., Zhang, B., Qi, C., Peng, H., Tsay, H.C., Soon, C.F., et al. (2022). Multi-Omics Integration Reveals Only Minor Long-Term Molecular and Functional Sequelae in Immune Cells of Individuals Recovered From COVID-19. Front Immunol *13*, 1–15. 10.3389/fimmu.2022.838132.
